# Evidence of multiple coordinate representations during generalization of motor learning

**DOI:** 10.1007/s00221-014-4034-6

**Published:** 2014-09-24

**Authors:** Pritesh N. Parmar, Felix C. Huang, James L. Patton

**Affiliations:** 1 University of Illinois at Chicago, Chicago, IL 60607 USA; 2 Sensory Motor Performance Program (SMPP), Rehabilitation Institute of Chicago, 345 E. Superior Street, Suite: 1406, Chicago, IL 60611 USA

**Keywords:** Motor learning, Adaptation, Intra- and inter-manual transfer, Visuomotor perception, Generalization and transfer, Internal model, Coordinate system, Neural representation

## Abstract

Several studies have suggested that the motor system takes advantage of a coordinate system when learning a novel sensorimotor environment. Such investigations, however, have not distinguished between initial preferences of a coordinate system versus possible changes due to learning. Here, we present experimental methods that specifically entertain the possibility of multiple coordinate systems during generalization. Subjects trained with their right arm on a viscous force field. We evaluated their performances for both arms in an untrained workspace before and after training using three fields, each representing extrapolation with a candidate coordinate system. Surprisingly, our results showed evidence of improvement (pre to post) in all fields for both limbs. These findings are consistent with the hypothesis of multiple, simultaneous coordinate systems involved in generalization. We also investigated how feedback might affect the results and found in several cases that performance was better for visual displays that were aligned with the limb (in first person) versus non-aligned.

## Introduction

A prominent question in human motion control is what coordinate system the brain uses to store and recall memories of successful actions. If such a coordinate reference frame is known, it would enable the ability to model and predict how some training situations may be better than others for a wide range of training applications that include sports coaching, flight simulation, and neurorehabilitation. For example, if the brain uses joint angles as the basic coordinate frame for representing action, this might suggest that the therapist should align their body in parallel with the patient as they provide therapeutic guidance.

Previous studies have suggested that our brain makes use of an intrinsic, joint-based coordinate reference when extrapolating learned skills (intra-manual transfer) to an untrained workspace with the training arm (Lackner and Dizio 1994; Shadmehr and Mussa-Ivaldi 1994; Gandolfo et al. 1996; Goodbody and Wolpert 1998; Shadmehr and Moussavi 2000). However, the results are ambiguous whether the observed success using an intrinsic reference frame is merely due to the learning or an advantage due to preexisting skills. An alternative evaluation is to focus on the pre-to-post performance changes due to learning. Here, we present a variant experimental technique that evaluates pre-to-post changes to determine whether one or more coordinate reference frame benefits from training. Our method contrasts against previous works that only considered whether a single, particular coordinate frame better explained experimental results.

In contrast to the intra-manual transfer, another practical situation requires a strategy to transfer skills between hands (inter-manual transfer). Previous studies have reported that the inter-manual transfer employs an extrinsic coordinate frame (Dizio and Lackner 1995; Criscimagna-Hemminger et al. 2003; Burgess et al. 2007). However, their methods only distinguished between the possibility of Cartesian and mirror representations without testing if there were others. Furthermore, it is puzzling that the intrinsic coordinate frame is favored by the trained arm, while the extrinsic coordinate frame is favored by the untrained arm. To date, no study has tested extrapolation of learned skills to an untrained workspace with an untrained arm. Several outcomes may result from such a “dual” transfer condition where one has to extrapolate and change hands. One possibility is that such transfer may result in interference of two representations and thus a degradation of performance. On the other hand, it may result in a winner-take-all phenomenon where one coordinate system is favored. Finally, it may result in several coordinate systems used simultaneously, suggesting multiple parallel representations contributing to the learned control.

Furthermore, research suggests that the manner in which feedback is presented may lead to different coordinate representations (Ghez et al. 2000). Absolute performance can be influenced differently by feedback that may be aligned (in first person) with the limb where cursor is projected directly above hand, than by a non-aligned, vertical display where cursor is projected on a computer screen. Increased movement variability and initial direction errors in aiming tasks have been reported when using a non-aligned display versus an aligned display (Bedard and Proteau 2005; Bo et al. 2006; Veilleux and Proteau 2011). While these studies highlight the impact of visual alignment on online control and movement planning, it is unknown how the display mode might influence the transfer of learned skills.

Our study focuses on evaluating generalizations of motor skills to an untrained workspace using both the training arm and non-training arm. We observed overall improvements in the untrained region and in both the end-point and joint-based fields, suggesting multiple, simultaneous coordinate representations. Furthermore, the display alignments (aligned vs. non-aligned) did not affect these observed performance improvements in the untrained workspace. Portions of this work have been presented in preliminary form (Parmar et al. 2011).

## Materials and methods

### Subjects

Thirty right-handed human subjects (20 M, 10 F) were recruited in this study after obtaining written informed consent approved by the local ethics committee. These subjects had no history of neurological, shoulder, or elbow disorders and were within the age range of 21–40 years. We excluded subjects with ambidexterity.

### Experimental setup

**Fig. 1:**
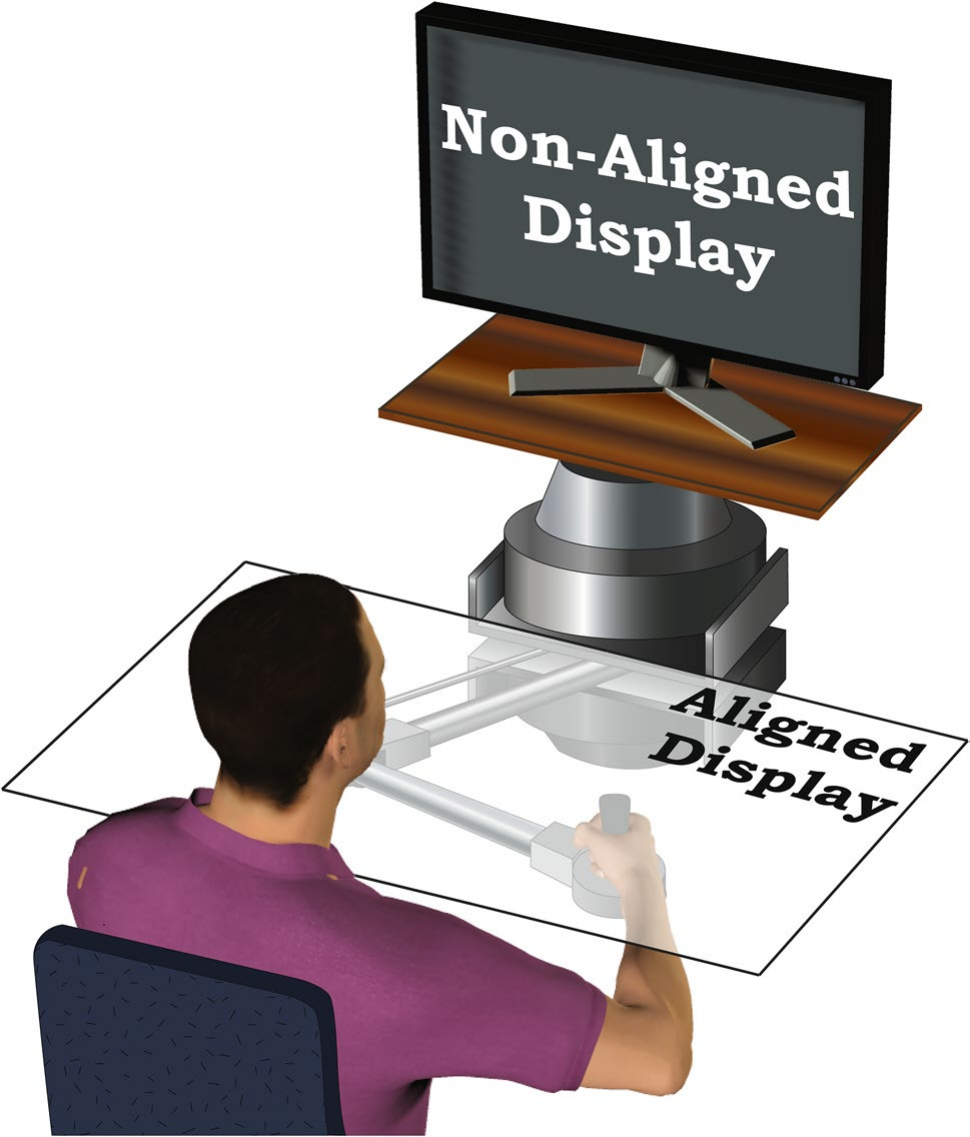
Sketch of the manipulandum, visual workspaces, and experimental setup. Subjects sat in front of the manipulandum robot and grasped the handle to perform the reaching task. The reaching targets were presented using either the aligned or the non-aligned display (Here, the aligned display is semi-transparent only for illustration purpose)

Subjects sat in front of a manipulandum robot and grasped the handle or the end effector (Fig. 1). We restrained their shoulders and supported their elbows by multi-link arm supports (JAECO/Rancho MultiLink Mobile Arm Support) so that their arm movements were planar. These multi-link arm supports had three degree of rotational freedom in a plane and had significantly low inertia compare to human arm.

The manipulandum was a light weight, low friction, two degrees of rotational freedom robot (Fayé 1986). The manipulandum was designed for clinical and neurorehabilitation research applications and was configured through impedance control for safe, stable, and compliant operation. Two low-inertia direct current torque motors (PMI Corp. model JR24M4CH, Kolmorgen Motion Technologies, Commack, NY) were mounted on the base of the robot and were connected independently to each joint using a parallelogram arrangement. Position measurements (400 Hz) were taken using two optical encoders (model 25/054-NB17-TA-PPA-QARIS, Teledyne Gurley, Troy, NY).

Each subject experienced one of two video displays (Fig. 1). These displays were used to show the position of the handle (as a cursor) and targets. The non-aligned display was a LCD monitor and mounted directly above the robot, approximately centered at eye level. The aligned display was an opaque, rectangular white screen, mounted horizontally above the handle of the robot. We used ceiling mounted video projector to project visuals on the aligned display. We calibrated both the aligned and non-aligned displays to represent the absolute spatial workspace of the handle.

### Experimental procedures

The reaching task was performed in one of two 20 × 20 cm^2^ workspace regions named *training* and *test*. Each workspace was centered 31 cm anterior to the chest, with the training workspace 30 cm to the right of the sagittal midline and the test workspace centered on the midline. In order to prevent inertial artifacts of the robot associated with changing the operating configuration, the workspaces were appropriately placed by moving each subject’s seat.

Each subject was instructed to move the handle of the robot to bring a cursor into a circular target. The cursor was 2-mm-diameter circle, which indicated the position of the handle, and the target was 1-cm-diameter circle. The reaching task included moving the cursor from the center of a workspace to a target and back (center-out reaching movements). Targets were placed 10 cm away from the workspace center in the direction of 24, 114, 204, and 294 degrees from *x*-axis. All subjects experienced the same random sequence of reaching targets chosen from the set of four movement directions. Furthermore, the number of different movement directions per phase experienced was the same for each subject.

During certain phases of the experiment, we programmed the manipulandum to produce velocity dependent forces at the handle, indicated by the vector *f* in the following equation:

1 $$ f = B\dot{x}, $$

where $\dot{x}$ was the velocity vector of the subject’s hand, and *B* was a constant viscosity matrix of the viscous environment:

2 $$ B = \left[ {\begin{array}{*{20}c} { - 15.69} & {9.80} \\ {9.80} & {15.69} \\ \end{array} } \right]{\text{N}} \cdot \text{s} /{\text{m}} . $$

As the subjects made reaching movements, forces were applied on their hand by the robot based on Eq. (1). Note that this field was translation-invariant in the end-point coordinates (*end*-*point field*).

During other phases of the experiment (explained below), we programmed the manipulandum to produce force fields which depended upon the angular velocities of the subject’s elbow and shoulder joints:

3 $$ \tau = W\dot{q}, $$

where *τ* was the torque vector acting on the subject’s shoulder and elbow joints, $\dot{q}$ was the subject’s joint angular velocity, and *W* was a constant viscosity matrix of the viscous environment in subjects’ joint coordinates. Note that the torque field described by Eq. (3) was translation-invariant in the *joint* coordinates, unlike the end-point field. The torque field in Eq. (3) could be converted to equivalent force field:

4 $$ f = \left( {J\left( q \right)^{\text{T}} } \right)^{ - 1}  W\dot{q}, $$

where $J\left( q \right) = \partial x/\partial q$ is the configuration-dependent Jacobian which maps the configuration from *q* to *x*, and the superscript *T* suggests the transpose operation. This illustrates how the force field defined by Eq. (4) depends on each subject’s limb geometry, origin, and current location in the workspace. We chose *W* such that the force field produced by Eq. (4) was equivalent to the end-point field in the training workspace. For each subject, the matrix *W* was calculated as the following:

5 $$ W_{\text{R}} = J_{\text{o,R}}^{\text{T}}  B J_{\text{o, R}} \;{\text{and}} $$

6 $$ W_{\text{L}} = J_{\text{o,L}}^{\text{T}}  B J_{\text{o,L}} , $$

where *W*_R_ and *W*_L_ are the joint-viscosity matrices, and *J*_o,R_ and *J*_o,L_ are the Jacobians evaluated at the center of the training workspace for the subjects’ right and left limb, respectively.

**Fig. 2:**
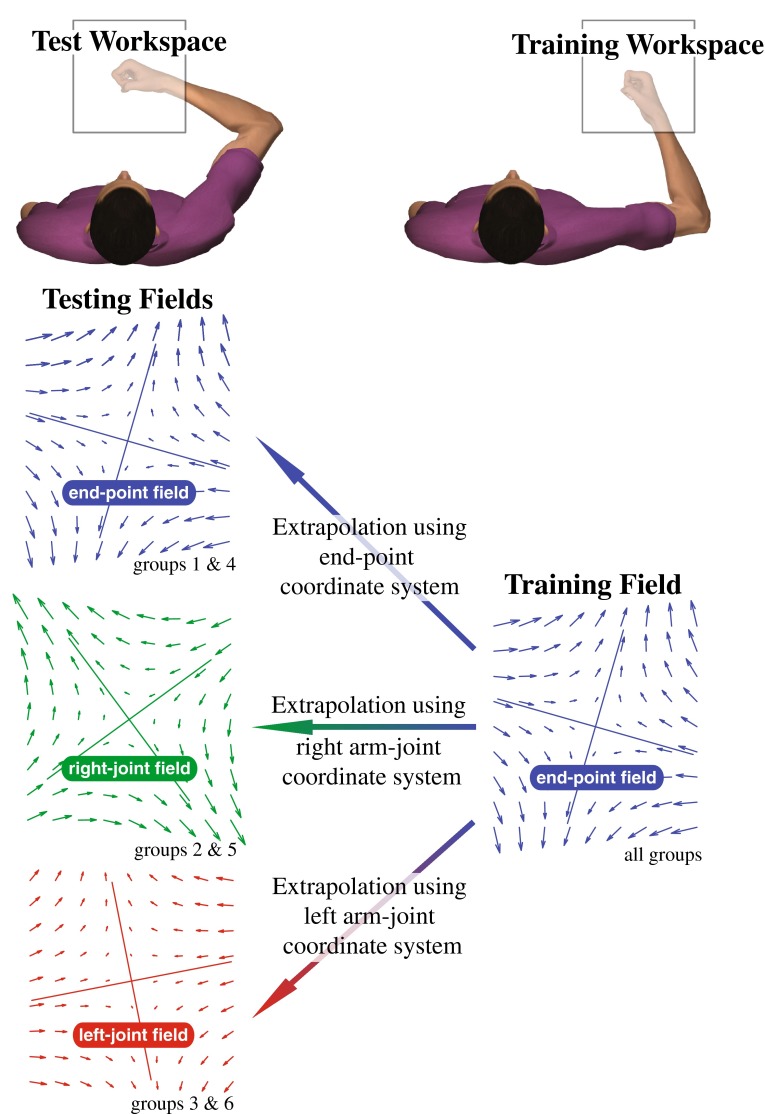
Velocity-dependent haptic force environments at the training and test workspaces. All groups were trained to perform the reaching task in the end-point field at the training workspace, and they were evaluated at the test workspace with a force environment, which was extrapolated using either the end-point, right-arm joint or left-arm joint coordinate system (*Solid lines* on vector fields represent eigenvectors)

The force fields produced using Eq. (4) with *W*_R_ and *W*_L_ hereafter termed the *right*-*joint field* and the *left*-*joint field*, respectively, which depended upon workspace location. At the training workspace, these force fields were identical to that produced by Eq. (1). However, this was not the case at the test workspace. Therefore, these testing fields could be viewed as the extrapolation of the training environment as illustrated in Fig. 2.

We trained all subjects to make reaching movements in the end-point field at the training workspace and subsequently evaluated their performance in either the end-point field, right-joint field, or left-joint field at the test workspace. The visual feedback of the hand position and target was provided using either the aligned or the non-aligned display. Hence, we define six distinct groups (3 types of test fields × 2 types of displays) with five subjects per group. We minimized speed variance with a slider graphic on the top of the screen that indicated satisfactory movement times within 0.4–0.6 s. To minimize the impact of learning during perturbations, the cursor position was removed for some trials (*no*-*vision trials*) after movement onset, detected using a speed threshold of 5 cm/s.

**Fig. 3:**
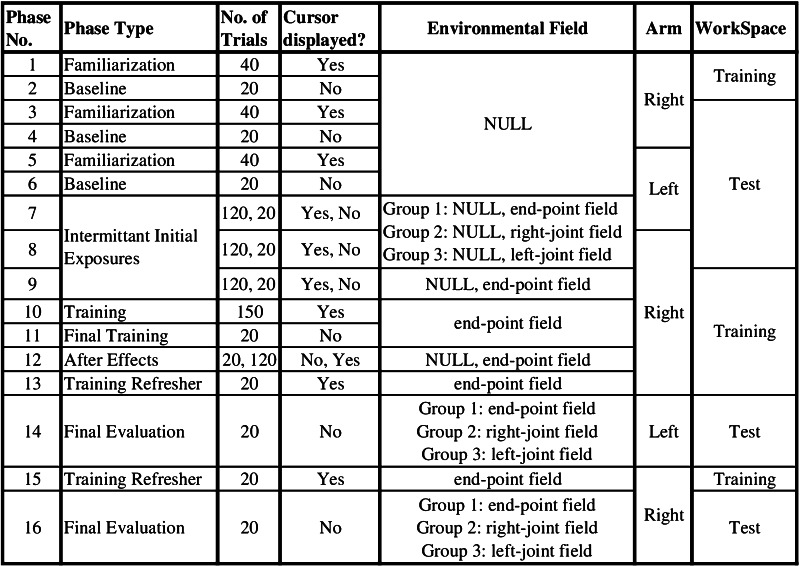
Summary of experimental procedure. Three groups underwent procedures as illustrated in this figure and received visual feedback using the aligned display. We repeated the same procedure for another three groups who received visual feedback using the non-aligned display

The experiment consisted of a number of distinct phases in order to comprehensively test all hypotheses (Fig. 3). We first assessed all subjects’ baseline performance (20 no-vision targets) after short familiarization (40 targets) for right limb at the training workspace and for both limbs at the test workspace. Then at the test workspace, we intermittently (20 out of 140 no-vision targets) assessed their both limbs’ initial performance in the presence of one of the three evaluation fields (depending on their group) for later comparison. These intermittent perturbation trials were distributed randomly throughout the total number of trials. Subjects could not anticipate the presence of perturbations because (1) force was proportional to velocity and hence only present after movement began and (2) visual feedback was removed only after movement onset. In the similar manner, the right limb’s initial performance in the training field (end-point field) was assessed at the training workspace. The training phase began with all groups practicing 170 movements in the end-point field using their right limb at the training workspace. Finally, the training limb was intermittently exposed to the null field, which assessed aftereffects of adaptation.

Our major objective was to assess the generalization of learning evidenced by the changes in performance in untrained region (test workspace). After a short training refresher at the training workspace (20 targets), we asked subjects to reach with their left and then right limbs (20 no-vision targets each) at the test workspace and in the presence of the evaluation field assigned to their group.

We also compared observed reaching movements of human subjects with ideal simulation models that generalize using a single coordinate system. Since the subject’s arm movements were restricted within a plane and their shoulders were restrained, the 2-link planar manipulator offered a close approximation. The dynamics were based on (cf. Murray et al. 1994):

7 $$ M\left( q \right)\textit{\"{q}} + C\left( {q,\dot{q}} \right)\dot{q} + N\left( {q,\dot{q}} \right) = \tau $$

where *τ* is the torque vector, *M*(*q*) is the inertial matrix, $C\left( {q,\dot{q}} \right)$ is the Coriolis matrix, and $N\left( {q,\dot{q}} \right)$ is the torques due to external environmental forces. In configuration space, the array of joint angles are expressed as *q* with its first and second time derivatives, $\dot{q}$ and $\textit{\"{q}}$.

The controller for this model was based on feed-forward and feedback schemes. The goal here was to produce joint torques such that the end-effector tracks a desired trajectory, *q*_*d*_. Given the dynamics of the system and *q*_*d*_, we wished to achieve *q*(*t*) = *q*_*d*_(*t*). We define the controller as following:

8 $$ \tau = M\left( {q_{\text{d}} } \right)\textit{\"{q}}_{\text{d}} + C\left( {q_{\text{d}} ,\dot{q}_{\text{d}} } \right)\dot{q}_{\text{d}} + N\left( {q_{\text{d}} ,\dot{q}_{\text{d}} } \right) + K_{\text{P}} \varepsilon + K_{\text{V}} \dot{\varepsilon } $$

where *M* and *C* terms computed using *q*_*d*_ represents the expectations of body’s own dynamics, and $K_{\text{P}} \varepsilon ,  K_{\text{V}} \dot{\varepsilon }$ represents feed-back strategy based on error difference between *q*_*d*_ and *q*. Here, *q*_*d*_ was assumed to be equal average baseline trajectory for a single typical subject. Also, *N* term in the controller represents expectation for the environmental forces that was based on a specific coordinate system. Hence, we define three ideal simulation models which use either the extrinsic coordinates system (Eq. 1), intrinsic coordinate system based on right arm (Eqs. 4, 5), or intrinsic coordinate system based on left arm (Eqs. 4, 6). Each of these three simulation models underwent the protocol (Fig. 3) and experienced each of the evaluation fields per simulation, resulting in nine different simulations. Expectation of the environmental forces (*N* term) was set to zero during the pre-training phases and assumed to be a perfect representation of the training force field based on each model’s respective coordinate system during the post-training phases.

The manipulator shoulder locations were chosen approximately to that of a typical subject with respect to the training and the test workspaces. The mechanical parameters (Shadmehr and Mussa-Ivaldi 1994) used in these mathematical models for a typical subject are provided in Table 1. The stiffness and viscosity matrices were acquired from the subjects’ initial exposure to the training field at the training workspace through least squares optimization. The dynamic equation was solved using ode23 in MathWorks MATLAB 2012a.

**Table 1:** Simulation model mechanical parameters

Upper arm	
Mass	1.93 kg
Inertia	0.0141 kg m^2^
Length	0.33 m
Center of mass	0.165 m
Forearm	
Mass	1.52 kg
Inertia	0.0188 kg m^2^
Length	0.34 m
Center of mass	0.19 m
Shoulder–shoulder length	0.40 m
Stiffness (*K*_P_)	$$ \left[ {\begin{array}{*{20}c} {20.7} & {6.6} \\ {6.6} & {21.4} \\ \end{array} } \right]{\text{N}} \, {\text{m}}/{\text{rad}} $$
Viscosity (*K*_V_)	$$ \left[ {\begin{array}{*{20}c} {2.7} & {1.1} \\ {1.1} & {3.1} \\ \end{array} } \right]{\text{N}} \, {\text{m}} \, \text{s} /{\text{rad}} $$

### Data analysis

To remain consistent with the previous work (Shadmehr and Mussa-Ivaldi 1994), we used the originally published performance measure, velocity correlation, as our primary metric. This measure quantified similarity between the velocity profile of individual hand trajectory and the corresponding velocity profile of average baseline trajectory (calculated per direction per workspace per subject). In order for subjects to show any improvement with this metric in a perturbing force field, they would have to learn appropriate compensation for timing as well as spatial deviation throughout movement. Trajectories were aligned using 5 cm/s onset speed threshold, and the correlation coefficient was computed using:

9 $$ \rho = \frac{{{\text{Cov}}(U,Y)}}{\sigma \left( U \right)\sigma \left( Y \right)} $$

where *ρ* is the correlation coefficient comparing *U* and *Y* velocity vectors. Here, *U* = (*u*_*1*_ *, u*_*2*_,…,*u*_*n*_) and *Y* = (*y*_*1*_ *, y*_*2*_,…,*y*_*n*_) are time series velocity vector sets. As Eq. (9) indicates, the correlation coefficient is a ratio between the covariance of time series and the product of their standard deviations. The value of *ρ* ranges between −1 and 1, which indicates perfectly positive and perfectly negative correlation, in this case between two time series of velocity vectors. A value of 0 implies orthogonal relationship. The covariance and standard deviation are defined as:

10 $$ {\text{Cov}}\left( {U,Y} \right) = \epsilon \left( {\left\langle {U - \epsilon \left( U \right),Y - \epsilon \left( Y \right)} \right\rangle } \right), $$

11 $$ {\text{where}} \;\left\langle {U,Y} \right\rangle = u_{i} \cdot y_{i} \;{\text{and}}\; \epsilon \left( {\left\langle {U,Y} \right\rangle } \right) = \frac{1}{n}\mathop \sum \limits_{i = 1}^{n} \left\langle {U,Y} \right\rangle $$

12 $$ \sigma \left( U \right) = \sqrt { \epsilon \left( {\left\langle {U - \epsilon \left( U \right),U - \epsilon \left( U \right)} \right\rangle } \right)} . $$

The $\epsilon$ operator represents the expected value of the argument and the symbol · in Eq. (11) indicates the dot product operation between two vectors.

**Fig. 4:**
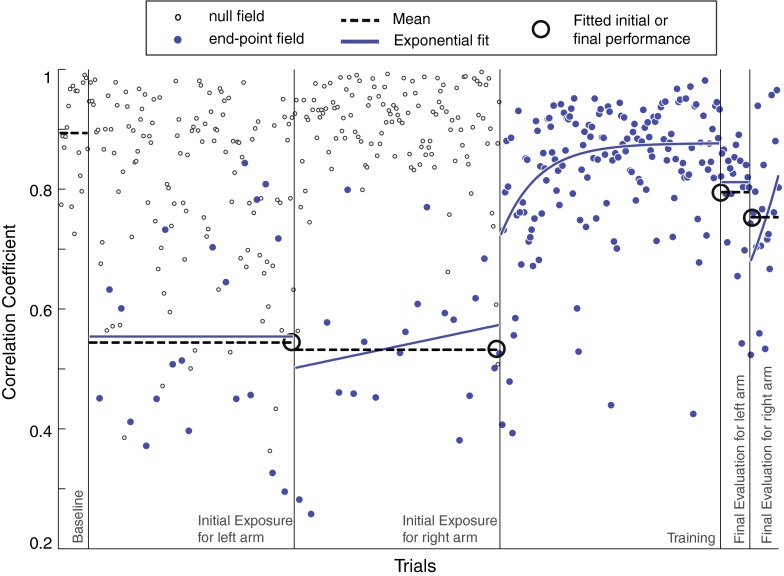
Course of performances at the test workspace (untrained region) in baseline, initial exposure, and final evaluation phases for an example subject (from end-point field, non-aligned display group). Linear regression failed to yield non-zero slopes for left and right arm initial exposure phases and left and right arm final evaluation phases (*p* = 0.43, 0.12, 0.36, and 0.16, respectively). Thus, the level of performance for the initial exposure and final evaluation phases were approximated to mean values (*big open circles*). Exponential regression for the training phase had root-mean-squared error of 0.09

In such an experiment with intermittent exposure to perturbations, it was possible that subjects might learn during the initial exposure and final evaluation phases at the test workspace (see Fig. 4). Researchers stated that intermittent experience of the force fields might be sufficient to learn its structure (Braun et al. 2009). Learning effects during these phases may be attributed to aspects that were common to all experimental conditions and/or learning of the perturbations. Because we are interested in evaluating pre-to-post improvement, one strategy for analysis might be to determine the difference between the last perturbed trial of initial exposure and the first trial of the final evaluation. However, because of the natural variability between perturbation trials, we concluded that regression intercepts would provide a better approximation of the true level of performance. We first tested for trends within the initial exposure and final evaluation phases (per subject). In the case when linear regression failed to yield a nonzero slope (*t* test at the 5 % significance level), we simply used the mean. However, when linear fit yielded nonzero slope, we fitted performances to an exponential regression:

13 $$ \widehat{\rho } = A + Be^{{ - {\text{trial}}/C}} . $$

Only the perturbed trials were used for these regressions. This approach removed any effect of learning that might have occurred within initial exposure or final evaluation phases. While linear regression determined trends, the exponential captured the typical nonlinear shape of learning curve tendencies during these phases. Note that an exponential function not only can imitate the saturation effects associated with learning, but also can represent linear trends if the data has such form.

Fitting exponential curves on the intermittent initial exposure performances (velocity correlation metric) allowed us to conservatively approximate subjects’ performance level at the test workspace just before the training (exponential curve fit value at the last trial number). Similarly, fitting exponential curves on the final evaluation phase allowed us to conservatively approximate subjects’ performance at the test workspace immediately after training (exponential curve fit value at the first trial number). This method permitted us to attribute any pre-to-post performance changes at the test workspace to the training at the training workspace. Note that the values for coefficient of determination for regression averaged 0.34, with root-mean-square error of 0.13. These values indicate only moderate fit, which was not surprising due to sparsity and variance in the data due to the unpredictable and intermittent nature of perturbations during these phases of the experiment.

### Statistical analysis

We analyzed the initial and final absolute performances at the test workspace using a repeated-measure analysis of variance (rm-ANOVA) with factors: arm (right and left), evaluation field (end point, right joint and left joint), display (aligned and non-aligned), and time (pre and post). Post hoc pairwise comparisons were evaluated using Bonferroni–Holm method.

In order to eliminate any confounding effects from differing initial conditions, we also performed a secondary analysis where each subject’s final evaluation performance was normalized by initial exposure performance. Pre-to-post changes for the normalized performances were analyzed using a 3-way analysis of variance (3-way ANOVA) with factors: arm, evaluation fields, and display. Furthermore, we compared the normalized pre-to-post changes against zero for each evaluation field using Bonferroni–Holm method.

## Results

**Fig. 5:**
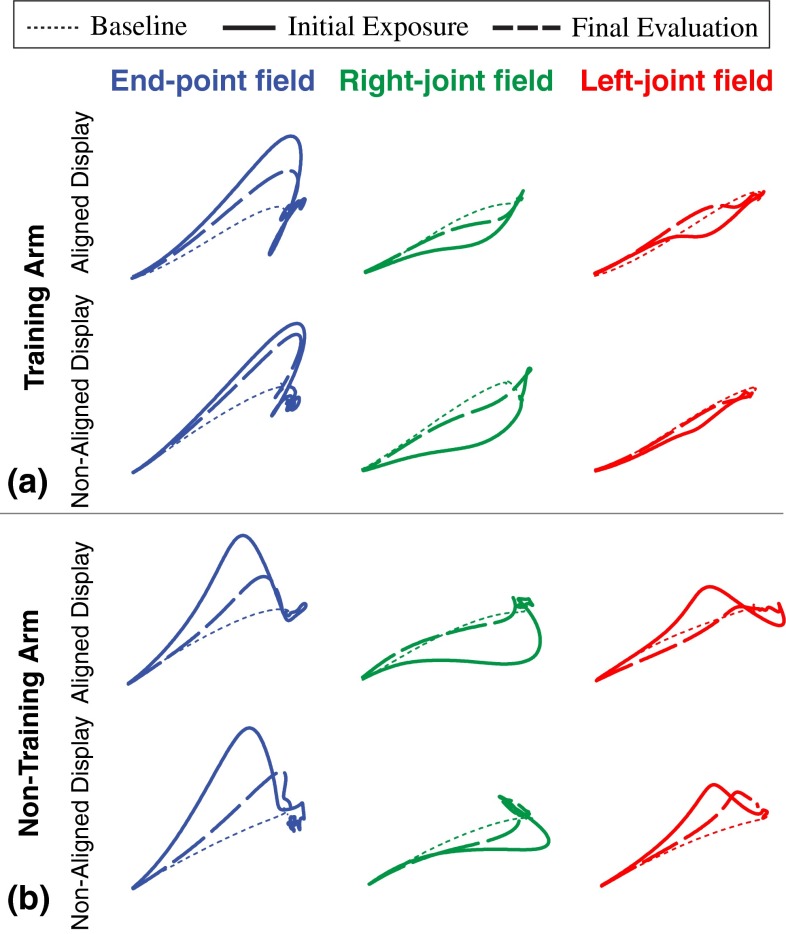
Group average hand trajectories at the test workspace (untrained region) for a representative target direction. Overall, the final evaluation trajectories are closer to baseline than the initial exposure trajectories for the training (**a**) and non-training arm (**b**)

As expected, baseline trajectories approximated straight lines to the target with nearly symmetric and smooth velocity profiles, regardless of limb, workspace, or visual display. All groups’ baseline motions were comparable, and correlation coefficients for right and left limb baseline averaged 0.94. Also typical of motor adaptation experiments, initial exposure to forces caused substantial error (Fig. 5) followed by corrective sub-movements that varied depending on the force field and location of each target. Figure 7a, c illustrates all groups’ initial performances in three different evaluation fields at the test workspace.

Across training, we observed characteristic initial errors that diminished as subjects practiced. By the end of training (in the training workspace, before transfer tests), groups did not differ in performance (*p* > 0.05). When the force field was intermittently removed, we observed aftereffects (average correlation coefficient = 0.78; *t* test comparison against baseline performance yielded *p* = 1.63e−19) consistent with numerous other studies (Shadmehr and Mussa-Ivaldi 1994; Gandolfo et al. 1996; Shadmehr and Moussavi 2000; Malfait et al. 2002), suggesting the presence of an internal model that predicts the environmental dynamics.

**Fig. 6:**
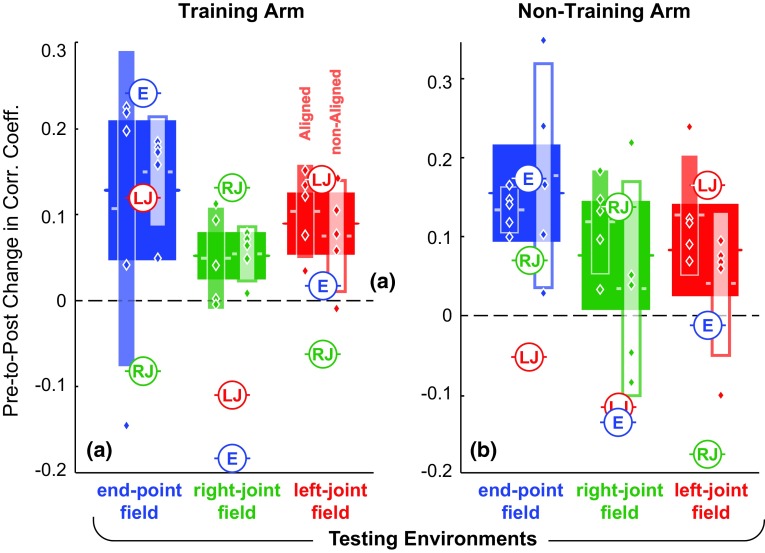
Summary of the pre-to-post performance changes for the training (**a**, right arm) and non-training arm (**b**, left arm) at the test workspace (untrained region). The performance significantly improved for all groups for intra- and inter-manual transfers (compared thick bars to zero). (The *thick bars* represent combined mean and 95 % confidence interval range for the groups they overlap. The thin bars represent individual group mean and 95 % confidence interval range. *Filled bar* = aligned display; *unfilled bar* = non-aligned display; *blue*, *green*, and *red color* = end-point, right-joint, and left-joint field; diamonds represent subjects’ individual contribution to their group. *Circles* show changes predicted by ideal models that extrapolate the training environment using either the end-point(*E*), right-joint (*RJ*) or left-joint field (*LJ*))

The major findings of this study involved the “test” workspace, where all groups exhibited overall improvement in performance over pre-training phases (rm-ANOVA; main effect of time; *p* = 3.42e−5). The levels pre-to-post changes in performance at the test workspace are shown in Fig. 6. Note, 95 % confidence interval ranges of pre-to-post change for the combined groups (who received the same evaluation fields) do not overlap zero for both arms.

**Fig. 7:**
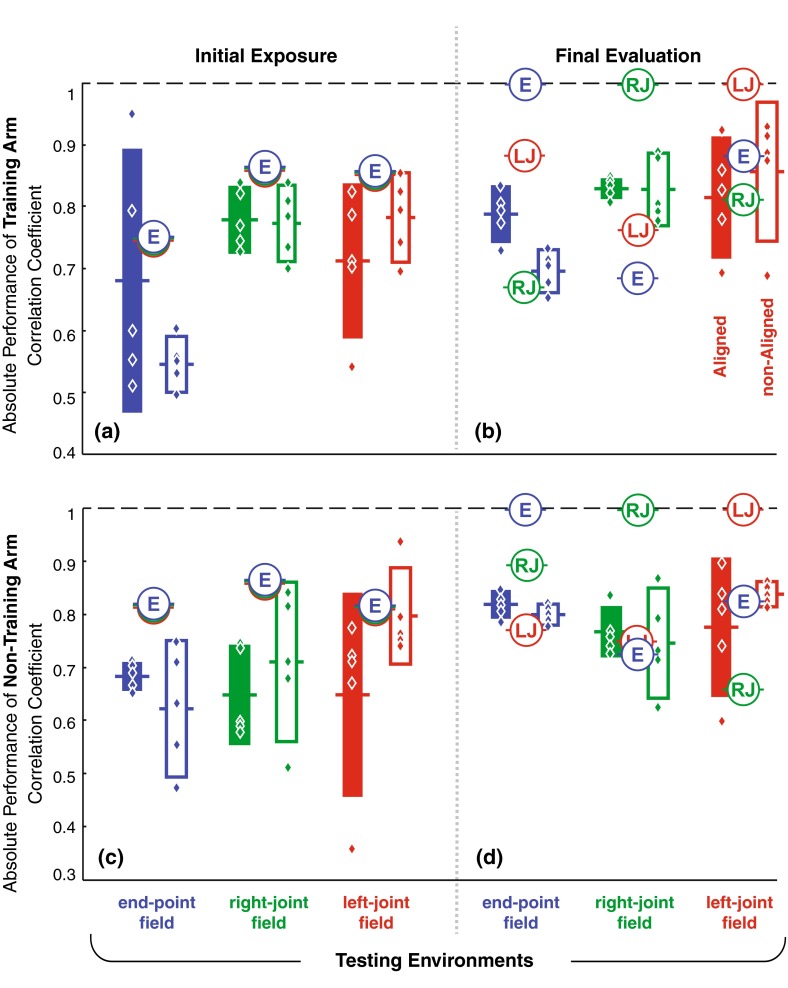
Summary of the absolute performance at the test workspace (untrained region): **a**, **b** initial and final evaluation for the training arm (right arm); **c**, **d** initial and final evaluation for the non-training arm (left arm). Performances in all evaluation fields significantly improved from initial exposure to final evaluation. There was also a higher absolute performance for the aligned display compared to the non-aligned display. Performance predicted by the ideal models spanned the range of subjects’ performance. (*Bars* represent individual group mean and 95 % confidence interval range. *Filled bar* = aligned display; *unfilled bar* = non-aligned display; *blue*, *green*, and *red color* = end-point, right-joint, and left-joint fields; *diamonds* represent subjects’ individual contribution to their group. *Circles* show changes predicted by ideal models that extrapolate the training environment using either the end-point (*E*), right-joint (*RJ*) or left-joint field (*LJ*))

There was a difference in initial exposure performances across different evaluation fields and display types (Fig. 7a, c), and so, we also inspected a normalized score by dividing each subject’s final evaluation by their initial exposure performance. These results were similar in showing overall pre-to-post improvements (3-way ANOVA followed by Bonferroni–Holm post hoc evaluations showing normalized change greater than zero; *p* = 2.60e−5, 2.11e−3 and 1.11e−3 for the end-point, right-joint and left-joint fields, respectively). Importantly, this second analysis revealed group differences in change across practice and showed a highest improvements for the group experiencing end-point field (3-way ANOVA; main effect of evaluation fields; *p* = 1.01e−2 followed by a Bonferroni–Holm comparisons; *p* = 1.45e−2).

Regardless of changes across practice, different field types had differing effects on performance. We saw lower performance in the end-point field compared to left-joint field in the non-aligned display condition (rm-ANOVA, interaction effect of evaluation fields and display followed by a Bonferroni–Holm comparison; *p* = 1.21e−4). There was a clearer lower performance for the end-point field when inspecting the right arm only (rm-ANOVA; interaction effect of evaluation fields and arm; Bonferroni–Holm comparisons; *p* = 2.80e−3 and 3.69e−2, for the right-joint and left-joint fields, respectively). Overall as expected, the right (practice) arm also performed better than the left, but this difference was detected only in the right-joint field (rm-ANOVA; interaction effect of evaluation fields and arm; followed by Bonferroni–Holm post hoc evaluations; *p* = 1.47e−2).

There was also difference between aligned over non-aligned displays. We found lower performance with the non-aligned display, but only for the end-point field versus left-joint field (rm-ANOVA; interaction effect of evaluation fields and display; Bonferroni–Holm comparisons; *p* = 1.21e−4).

We formulated three ideal models to provide a theoretical framework for understanding how these generalizations might have occurred. These models reached in straight lines toward the targets during the baseline phases, showed comparable initial errors when initially exposed to the force environments, and exhibited aftereffects in the training workspace after simulated learning had taken place. Importantly, each of the three idealized models used a differing coordinate representation to extrapolate (generalize) the learning to the test workspace. Each model approximately predicted initial exposure performance in the force fields (Fig. 7a, c), where each ideal model’s result was identical. However, during final evaluation in the test workspace, ideal model performances spanned the range of subjects’ performance (Fig. 7b, d). Note that performance in the final evaluation was at best when the model’s coordinate representation matched that of the evaluation field.

It should be noted that the ideal model based on a single coordinate system alone does not accurately predict the observed performance changes. If our subjects extrapolated their learned skills solely using an intrinsic (joint) coordinate system for the training arm, as suggested by others (Lackner and Dizio 1994; Shadmehr and Mussa-Ivaldi 1994; Gandolfo et al. 1996; Goodbody and Wolpert 1998; Shadmehr and Moussavi 2000), one would find results similar to that of the ideal model which extrapolates using right-arm joint coordinate system. However, this model predicted negative pre-to-post changes in the end-point and left-joint fields in contrast to observed changes for the human subjects. Furthermore, if our subjects extrapolated their learned skills solely using an extrinsic (Cartesian) coordinate system for the non-training arm (Dizio and Lackner 1995; Criscimagna-Hemminger et al. 2003; Burgess et al. 2007), one would find results similar to that of the ideal model which extrapolates using end-point coordinate system. However, this model also predicted negative pre-to-post changes in the right-joint and left-joint fields in contrast to observed changes for human subjects.

## Discussion

This study investigated, based on the pre-to-post performance changes, how well either limb might extrapolate skills to unpracticed parts of the workspace. A skill transfer paradigm with both intra-manual and inter-manual tests of generalization provided evidence to reveal the presence of multiple, simultaneous representations. As shown by the pre-to-post changes, performance improved for both end-point and joint-based fields in the untrained workspace.

Simulation results suggest that if subjects were learning a single coordinate representation, their movements would be incompatible with any evaluation fields used in our experiment. However, it stands to reason that if subjects are learning some form of a mixture of coordinate representations, there could be better overlap with the characteristics of the component environments, leading to success in skill transfer in all conditions, as we observed. Moreover, the performance changes observed for our subjects were within the range predicted for the performance change by all three ideal models, implying that there might be mixture of coordinate systems.

Our results are consistent with the hypothesis that multiple, simultaneous internal representations are used by the nervous system for sensorimotor control. These representations include extrinsic and intrinsic reference frames that are evident in the performance improvements in the untrained region for both intra- and inter-manual transfer evaluations. Such performance improvements were observed for all three evaluation conditions, each representing extrapolation with a candidate coordinate system. If subjects, for example, had developed the internal model only based on the intrinsic space, we would not have observed all of the performance improvements seen.

Such representations may each exist in distinct parts of the nervous system. Recently, Wiestler et al. (2014) purported that representations may exist not only in world-centered coordinates, but also in body-centered coordinates represented in the dorsal premotor cortex (PMd). Our results are also in line with recent work by Brayanov et al. (2012) using visual rotation adaptation, which suggested that representation of learning is based on a combination of local representations in intrinsic and extrinsic coordinates. Furthermore, Berniker et al. (2014) suggested that intra-manual generalization patterns were better accounted by a mixture of representations, or by non-parametric models that assumed local learning with graded, decaying generalization. The current work reaches similar conclusions, but focuses on evidence from pre-to-post changes, with interpretation from optimized dynamic simulations of the human arm. The natural structure of each sensory system may lend itself to the use of different coordinate representations. Krakauer et al. (1999) showed that hand kinematics is learned from visual errors in extrinsic coordinates, while dynamics are learned from proprioceptive errors in intrinsic coordinates. The eyes do not directly encode events in extrinsic coordinates, but their influence on sensorimotor control is more likely to be influenced by extrinsically represented system than any other. Thus, separate sensory modalities might be employed in constructing different internal models that are based on their respective coordinate systems.

This notion that vision is more *extrinsic* can also explain how display alignment affects performance. Because subjects simultaneously observed visual error (as a cursor) while they trained, one might expect that the aligned visual feedback condition would enable better performance, which was observed. More importantly, one might also expect that subjects’ performance in the intrinsic fields would not be as influenced by the type of visual feedback, which was also observed. This selective influence on performance may be the most compelling indication of how the relative strength of different coordinate representations might be altered by experimental conditions.

Other modeling and experimental studies support the concept of multiple simultaneous representations. Wolpert and Kawato (1998) proposed a possible mechanism for multiple paired forward and inverse models explaining partial generalization. It was postulated based on the assumption that a single neuron cannot learn to cope with all different types of dynamics and kinematics of the environment and objects. Based on this modular structure hypothesis, different modules can learn a task at the same time and build different internal models while training, and depending on feedback cues, they can be called upon independently or simultaneously during generalization. For example, researchers show that the CNS may encode simpler dynamics in extrinsic coordinates and more complex dynamics in intrinsic coordinates (Ahmed et al. 2008). Furthermore, a model employing two coordinate representations has been proposed, where a weighted sum of reference frames can be shaped by the context of the environment (Berniker and Kording 2008). These internal models can also have interference or combinatory effect during adaptation (Ghahramani and Wolpert 1997; Shadmehr and Brashers-Krug 1997; Blakemore et al. 1998; Wolpert and Kawato 1998; Flanagan et al. 1999; Haruno et al. 1999; Shadmehr and Holcomb 1999). We suspect that separate networks encode the inverse dynamics of the environment, based on both extrinsic versus intrinsic coordinates. While this study investigated three possible coordinate systems, there maybe no end to the possible coordinate representations used, and perhaps learned through experience, in the nervous system.

Findings on coordinate representations for inter-manual transfer have not been consistent. The literature suggests that inter-manual transfer prefers mirror-symmetrical coordinates for a typewriting task (Hicks et al. 1982), but this was not consistent with an inverted mirror printing task (Hicks 1974) which seem to utilize extrinsic coordinates. Furthermore, many studies have reported inter-manual transfer of dynamics in extrinsic coordinates (Dizio and Lackner 1995; Criscimagna-Hemminger et al. 2003; Burgess et al. 2007). These methods determined a winner without allowing for the possibility for multiple representations. Our application of a workspace generalization test, combined with inter-manual transfer and pre-to-post evaluations, provided an opportunity for the interpretation of multiple coordinates and may explain the varying results of previous studies.

This study was not exhaustive in that it only performed tests of dominant to non-dominant transfer. Others have shown that dominant to non-dominant transfer has an advantage in some cases (Parlow and Kinsbourne 1990; Gordon et al. 1994; Thut et al. 1996; Teixeira 2000), while not in others (Hicks 1974; Taylor and Heilman 1980; Parlow and Kinsbourne 1990). One explanation is that the dominant hemisphere controls both arms (Sainburg 2002) and hence is more involved than its opposite hemisphere (Geschwind 1975; Taylor and Heilman 1980; Kawashima et al. 1993, 1994; Dassonville et al. 1997; Viviani et al. 1998). It remains to be seen whether transfer would be the same in a non-dominant to dominant test.

As found by many other studies, movement performance seems to be affected by visual orientation. Video aiming tasks using a non-aligned display versus an aligned display have been reported to affect the performance: movement planning (Bo et al. 2006) and online control (Graham and MacKenzie 1996; Proteau and Isabelle 2002; Robin et al. 2005; Veilleux and Proteau 2010). Previous studies have reported increase in variability and error for non-aligned display (Bedard and Proteau 2005; Bo et al. 2006; Veilleux and Proteau 2011). Our results suggest that the display orientations do not seem to affect inter- and intra-manual transfer of haptic skills.

A sensory discrepancy might explain why performance was sometimes worse when using the non-aligned display. We argue that when hand position is presented visually on a non-aligned display, the CNS has to transform the locations presented on a vertical plane to a movement (in our case horizontal) plane, and the additional processing required for this transformation might affect performance. If proprioceptive and visual signals are not aligned, this may lead to increased uncertainty regarding the initial position of hand (Graham and MacKenzie 1996; Proteau and Isabelle 2002; Robin et al. 2005; Veilleux and Proteau 2010). In primates, bimodal neurons in the posterior parietal cortex receive proprioceptive and visual signals to accurately determine the position of the hand (Graziano et al. 2000). It is possible that activity in this area plays a role in coping with the disparity between sensory signals. One might predict that lesions in this area may dramatically influence the ability to coordinate actions when they involve transformations.

Although our method of probing the structure of internal model is similar to Shadmehr and Mussa-Ivaldi (1994), this study introduced new protocol, analysis, and conclusions. Their conclusions were based only on the final evaluation for intra-manual transfer (with non-aligned display), which suggested that the nervous system prefers intrinsic (joint) coordinates since the subjects’ absolute performance after training was better in the right-joint (intrinsic) field than in the end-point (extrinsic) field. However, performance in the presence of force fields based in different coordinate systems may simply differ overall. One striking finding from our experiment was that the subjects’ initial (before training) performance with non-aligned display in the right-joint and left-joint fields was significantly better than in the end-point field (Fig. 7a) even though force magnitude experienced were almost similar. This suggests that the joint fields were advantageous for subjects before the training had begun that could be reflective of dynamics or other aspects that were unrelated to learning. Our pre-to-post evaluations allowed for an understanding of actual improvement due to training that support learning in several frames of reference.

Experimental approaches are not always optimal and without confounding effects. One limitation may have been that our choice of performance metric did not fully capture all aspects of learning. We also performed the analysis using other metrics of performance (such as maximum perpendicular deviation and average error) but found more equivocal results. This was no surprise, however, because such measures lack sign that indicate direction of error, nor did they indicate error in extent. This is mainly due to the type of force field used in this study. These metrics failed to capture subjects’ performance when movements were closely aligned with eigenvectors of the evaluation fields. On the other hand, the correlation metric in the velocity domain effectively captured any variation in timing and lateral deviation from the average baseline velocity profiles.

Another limitation may have been that (unwanted) learning might have taken place during the evaluation phases of the experiment that were designated for testing. To minimize this confound, our exponential fits on the intermittent initial exposure and final evaluation phases allowed us to remove any learning effects. We also tested for fast change in performance between trial 1 and 2 and found that this was not significantly different from zero (*p* > 0.5).

One final speculation was on the actual ratio of the mixture of coordinate systems involved. We performed a “best fit” optimization on all intra-manual trajectories of all subjects, minimizing error between model and subject data. We found fairly consistent ratio of 0.31 ± 0.13 Cartesian, 0.62 ± 0.10 right-joint, and 0.18 ± 0.08 left-joint coordinate systems (mean ± 95 % confidence interval). These rough estimates indicated a bias toward right-joint coordinate systems, which may explain experiments that favored right joint (Shadmehr and Mussa-Ivaldi 1994). It remains to be seen in studies that put this ratio to the test for predicting transfer of learning.

This study provides evidence to support multiple coordinate systems when transferring learned skills to unpracticed environments. It remains to be seen whether these different representations are performed by different parts of the brain, but if so, lesions in specific areas might reveal the biasing of one representation in training over another. This impacts not only how we interpret the results from other motor control investigations, but may also guide the development of robotic neurorehabilitation. It may also suggest that machine-learning algorithms used to model patients (or any subject that is training) should embody a combination of multiple coordinate representations, which may inform more optimal training algorithms.
